# Personalized Medicine: Unraveling the Potential of Diamine Oxidase Deficiency

**DOI:** 10.3390/jcm13226797

**Published:** 2024-11-12

**Authors:** Hilario Blasco-Fontecilla

**Affiliations:** 1 Instituto de Investigación, Transferencia e Innovación (ITEI), Universidad Internacional de La Rioja (UNIR), 26006 Logroño, Spain; hilariomanuel.blasco@unir.net; 2 Department of Psychiatry, Emooti, 28010 Madrid, Spain; 3 Center of Biomedical Network Research on Mental Health (CIBERSAM), 28029 Madrid, Spain

The Special Issue Diamine Oxidase Deficiency: Prevalence, consequences, and solutions brings together a series of groundbreaking studies that explore the role of four single-nucleotide polymorphisms (SNPs) (rs10156191, rs1049742, rs1049793, and rs2052129) of the Diamine Oxidase (DAO) across various medical conditions, with a special focus on its influence on histamine metabolism. Histamine, a crucial biogenic amine, plays diverse roles in inflammatory responses, neurotransmission, and immune regulation. DAO is responsible for breaking down excess histamine in the body, and its deficiency can lead to histamine intolerance (HIT) and histamine-related disorders. This editorial provides an integrative overview of the articles featured in this issue, along with several other studies, and highlights future research directions.

One of the central themes in this issue revolves around the interaction between DAO and Attention-Deficit/Hyperactivity Disorder (ADHD). ADHD is the most frequent neurodevelopmental disorder, with a worldwide prevalence around 5% [1]. In a seminal work, Blasco-Fontecilla (2023) revisited the Speer allergic tension fatigue syndrome (SAFTS) and proposed that histamine, rather than acetylcholine, might be the missing link between ADHD and allergic conditions, among others [2]. He argued that histamine dysregulation, particularly in individuals with DAO deficiency, may contribute to the overlap between ADHD and allergies. In a subsequent pilot study in 303 children and adolescents diagnosed with ADHD, we provided crucial insights into the prevalence of DAO gene variants among children and adolescents with ADHD, showing that a significant proportion (79%) carried genetic markers associated with DAO deficiency [3]. Importantly, we reported that the homozygosity of the DAO SNPs variants 1 (rs10156191) and 4 (rs2052129) related to severe DAO deficiency was associated with a lower intelligence quotient (IQ) and, particularly, a much lower working memory (WM). This finding is intriguing as, theoretically, histamine does not cross the blood–brain barrier (BBB) [4]. Accordingly, the increased levels of histamine secondary to DAO deficiency should not influence brain functioning. However, recent research in animal models suggest that mast cell activation during inflammation mediates BBB impairment and cognitive dysfunction [5]. Lastly, Tobajas et al. (2024) investigated how psychostimulant drugs, widely used in ADHD treatment, interact with DAO [6]. Their findings suggest that two stimulants widely used in the treatment of ADHD, namely methylphenidate and lisdexamphetamine, do not interact with the DAO enzyme. Yet, the potential role of DAO supplementation in core ADHD symptoms remains unproved.

Okutan et al. (2023) presented a double-blind, placebo-controlled clinical trial on DAO supplementation in women with fibromyalgia [7]. This study is one of the first to provide evidence of the therapeutic benefits of DAO in managing the chronic pain and fatigue characteristic of fibromyalgia. The authors found that exogenous DAO supplementation significantly reduced pain in patients with HIT, underscoring the role of histamine in fibromyalgia symptoms. This study could pave the way for the development of new treatment protocols for patients with fibromyalgia, especially for those patients with DAO deficiency.

Tobajas et al.’s second paper in this issue built on these findings by investigating the interaction between DAO and psychotropic medications used in fibromyalgia treatment [8]. Interestingly, they found that DAO levels remained largely unaffected by most psychotropic drugs, with the notable exception of citalopram, which reduced DAO activity. These insights highlighted the importance of considering histamine metabolism when prescribing psychotropic medications for fibromyalgia, as patients with DAO deficiency might experience worsening symptoms due to histamine accumulation. In another work, Tobajas et al. (2023) also explored how DAO interacts with anti-inflammatory and anti-migraine drugs [9]. This study provided valuable insights into the metabolic interactions between DAO and drugs like non-steroidal anti-inflammatory drugs (NSAIDs) and triptans, commonly prescribed for migraines. The authors found that certain anti-inflammatory medications can inhibit DAO activity, leading to elevated histamine levels, which in turn may exacerbate migraine symptoms. This suggests that clinicians should consider histamine metabolism in patients who are unresponsive to standard migraine treatments, particularly those with DAO deficiency.

Finally, in a pioneering study, Ponce Díaz-Reixa et al. (2023) examined the relationship between genetic DAO deficiency and lower urinary tract symptoms (LUTS) [10]. This research was among the first to suggest that HIT could manifest as urinary symptoms, expanding the clinical spectrum of histamine-related disorders. By identifying a high prevalence of DAO deficiency in patients with unexplained LUTS, the authors proposed that histamine metabolism should be investigated as a potential underlying factor. This study opens new possibilities for treating LUTS by targeting histamine pathways and DAO function.

To sum up, it was quite interesting to find in that Special Issue that (1) most of the studies reported that around 80% of the affected patients, irrespectively of the studied pathology, had at least one defective DAO allele and (2) there is increasing evidence linking ADHD with the remaining somatic conditions studied in this Special Issue. Thus, ADHD is frequently comorbid with fibromyalgia [11,12], migraine [13,14], and LUTS [15,16]. Accordingly, it is tempting to speculate about a putative core, shared mechanisms that may be (why not?) histamine [2].

Gaps in the knowledge, clinical implications, and future directions.

The collective findings from these studies provide compelling evidence that DAO deficiency and histamine dysregulation play a central role in multiple medical conditions, ranging from ADHD and fibromyalgia to migraines and LUTS. While these studies have laid a strong foundation for understanding the importance of histamine metabolism, they also highlight the need for further research to refine diagnostic and therapeutic strategies. For instance, one of the recurring themes across these studies is the concept of personalized medicine. The variability in histamine metabolism due to genetic factors underscores the need for individualized approaches to diagnosing and treating conditions related to DAO deficiency. Testing for DAO activity and genetic markers could become a routine part of medical practice in conditions like ADHD, fibromyalgia, and migraines, where histamine-related pathways may be involved.

To date, the most relevant gaps in the knowledge concerning the impact of DAO enzymes on SNP variation that require further research are as follows:

1. Although studies in this Special Issue report a prevalence of around 80% for at least one allele associated with defective DAO enzyme functioning, there is still limited information on the prevalence of specific DAO enzyme alleles in the general, healthy population. Indeed, defective DAO alleles, which impact the breakdown of histamine, appear to be relatively common in the general population. However, these findings are primarily derived from control groups in case–control studies. For instance, one study comparing patients with HIT and healthy controls found that 79% of HIT patients and 72% of healthy controls carried one or more SNPs associated with reduced DAO enzyme activity [17]. This is unsurprising, given the ubiquity of histamine in most foods, suggesting that humans have evolved to exhibit a wide range of effectiveness of histamine metabolism.

2. The clinical study of alleles associated with defective DAO functioning is complicated by the potential for phenocopies, as DAO enzyme activity can be inhibited by various drugs [18,19,20,21,22] and influenced by clinical conditions like dysbiosis [23]. Interestingly, quercetin improved gut dysbiosis in antibiotic-treated mice [24].

3. The interaction between alleles of both DAO and the other histamine-metabolizing enzyme, histamine-N-methyltransferase (HNMT), is underexplored, particularly in diseases involving the central nervous system. A notable exception is in the case of migraines, where both enzymes appear to interact synergistically [25]. Ideally, studies on ADHD and other mental disorders should consider the role of both histamine-degrading enzymes, as it has been the case in several other pathologies such as asthma or colitis ulcerosa, among others [26,27,28,29,30,31,32,33].

4. HIT occurs when there is an imbalance between histamine intake and its degradation, often due to reduced DAO activity [34,35,36]. Dysbiosis, or gut microbiota imbalance, can exacerbate HIT by increasing histamine-producing bacteria, further overwhelming DAO’s capacity. This impaired histamine breakdown can result in symptoms such as gastrointestinal distress, headaches, and skin reactions. Dysbiosis is closely linked to HIT by both increasing histamine production and impairing DAO activity [23,37]. Additionally, the enteric nervous system (ENS), often referred to as the “second brain”, plays a crucial role in regulating digestion and communicating with the brain via the gut–brain axis. It contains approximately 500 million neurons, the same number as in a cat’s brain. Although its physical weight is less significant, its neural complexity is notable, with the ENS estimated to weigh around 1-2 kg, including the entire neural network embedded in the gut. This “second brain” significantly influences mood, digestion, and overall health, operating semi-independently from the central nervous system [38,39,40].

## Conclusions

The research presented in this Special Issue highlights the pivotal role of DAO in maintaining histamine balance and its far-reaching implications for health and disease. As we continue to uncover the complex interactions between DAO, histamine, and various medical conditions, it becomes increasingly clear that DAO deficiency is a significant factor in many chronic and difficult-to-treat conditions [41,42,43]. The future of DAO research holds promise for the development of new diagnostic tools, personalized treatment strategies, and novel therapeutic interventions that could transform the management of histamine-related disorders [44,45].

By advancing our understanding of DAO’s role in conditions like ADHD, fibromyalgia, migraines, and LUTS, these studies offer valuable insights that will shape future research and clinical practice. As we move forward, the integration of DAO testing into routine medical care and the development of histamine-targeted therapies may become key components of personalized medicine, improving patient outcomes across a wide range of conditions.

All these findings open new avenues for personalized ADHD treatments by targeting histamine metabolism. Future studies should aim to develop standardized protocols for assessing DAO activity and determining when DAO supplementation or dietary interventions might be beneficial. However, the intricate interplay of various factors influencing DAO alleles, including allele prevalence and phenocopies due to drug interactions or clinical conditions like dysbiosis, histamine levels, and the interaction between DAO and HNMT alleles, presents a complex challenge for researchers. These factors complicate the clinical understanding of histamine-related conditions, such as HIT and migraine. Given the complexity of these interactions, data science and artificial intelligence (AI) may be the most promising tools available to address these gaps comprehensively. AI can analyze vast datasets, uncover hidden patterns, and model the multi-dimensional relationships between genetic, environmental, and clinical factors, which traditional research methods may struggle to achieve. Accordingly, AI is more frequently used in complex medical problems resulting from the combination and interaction of massive datasets [46,47]. The articles published in this Special Issue are simply the starting point for research on the role of the DAO enzyme in ADHD, other neurodevelopmental disorders, and their comorbidities.
